# A method for estimating the oxygen consumption rate in multicellular tumour spheroids

**DOI:** 10.1098/rsif.2013.1124

**Published:** 2014-03-06

**Authors:** David Robert Grimes, Catherine Kelly, Katarzyna Bloch, Mike Partridge

**Affiliations:** The Gray Institute for Radiation Oncology and Biology, University of Oxford, Old Road Campus Research Building, Oxford OX3 7DQ, UK

**Keywords:** hypoxia, oxygen consumption, oxygen diffusion, tumour spheroids, mathematical modelling

## Abstract

Hypoxia occurs when oxygen levels within a tissue drop below normal physiological levels. In tumours, hypoxia is associated with poor prognosis, increased likelihood of metastasis and resistance to therapy. Imaging techniques, for example, positron emission tomography, are increasingly used in the monitoring of tumour hypoxia and have the potential to help in the planning of radiotherapy. For this application, improved understanding of the link between image contrast and quantitative underlying oxygen distribution would be very useful. Mathematical models of tissue hypoxia and image formation can help understand this. Hypoxia is caused by an imbalance between vascular supply and tissue demand. While much work has been dedicated to the quantitative description of tumour vascular networks, consideration of tumour oxygen consumption is largely neglected. Oxidative respiration in standard two-dimensional cell culture has been widely studied. However, two-dimensional culture fails to capture the complexities of growing three-dimensional tissue which could impact on the oxygen usage. In this study, we build on previous descriptions of oxygen consumption and diffusion in three-dimensional tumour spheroids and present a method for estimating rates of oxygen consumption from spheroids, validated using stained spheroid sections. Methods for estimating the local partial pressure of oxygen, the diffusion limit and the extents of the necrotic core, hypoxic region and proliferating rim are also derived. These are validated using experimental data from DLD1 spheroids at different stages of growth. A relatively constant experimentally derived diffusion limit of 232 ± 22 μm and an O_2_ consumption rate of 7.29 ± 1.4 × 10^−7^ m^3^ kg^−1^ s^−1^ for the spheroids studied was measured, in agreement with laboratory measurements.

## 1. Introduction

Medical imaging has advanced to the stage where it is now technologically feasible to incorporate positron emission tomography (PET)/CT information into radiotherapy planning. Strategies for using this information have been proposed that range from assisting definition of boost dose regions to the design of IMRT plans which spatially modulate dose voxel-by-voxel on the basis of the functional images, commonly referred to as dose painting [1–3]. However, these strategies involve significant departures from the current practice of delivering uniform population-based dose to the effective CT-derived treatment volume, and thus away from the clinical evidence base that informs current treatments. While the incorporation of functional imaging holds much promise for radiotherapy planning, for example, by selectively boosting regions of hypoxia, there is still a large amount unknown about the biology underpinning it and how to relate this to the images obtained.

Hypoxia is one of the major hallmarks of solid tumours; it occurs when the oxygen requirements of a tissue outstrip the ability of the local vasculature to supply oxygen. It has been known since the 1950s [4] that hypoxic tissue is more radioresistant than well-oxygenated tissue, and this factor has a large impact on a treatment [5], requiring higher levels of radiation to elicit the same cell kill. In the context of radiotherapy, the oxygen effect is quantified by the oxygen enhancement ratio, which is the increased effect of ionizing radiation on cell kill in the presence of molecular oxygen. This is taken as unity for anoxic cells and has been shown to be around 2.5–3 in well-oxygenated cells *in vitro. In vivo* tumours typically have heterogeneous oxygen distributions throughout. Imaging oxygen distribution in tumours has consequently been the focus of much interest [6,7]. PET with tracers, for example ^18^*F*-FMISO, are capable of mapping regions of hypoxia in an image [8]. However, this technology has a resolution of about 3–5 mm, whereas oxygen diffusion limits are typically 100–200 μm [9–11] and thus not possible to directly resolve. The question of how we translate an intensity distribution on a PET scan to a radiotherapy dose prescription remains largely unanswered.

In order to effectively plan irradiation of a tumour based on macroscopic scale images, it is important to understand oxygen gradients within a tumour and what impact these have on the image formation. Since the early 1900s, many mathematical descriptions of oxygen distribution in tissue have been proposed. Early mathematical models focused on the diffusion of oxygen into a cylindrical mass of tissue [9,12]. This was extended to estimate the diffusion from a cylindrical blood vessel [13]. Validating such models presents significant obstacles, as directly measuring oxygen concentration in tissue is not straightforward. As a result, most model testing has used histological sections stained for both hypoxia and vasculature to estimate the diffusion radius and compare this to the predicted limit [9,13]. One of the major limitations of this approach is that the presence of vasculature does not necessarily translate to a well-oxygenated blood supply, as tumour blood vessel functionality is temporally heterogeneous and tumour microvasculature is often chaotic and poorly perfused. For this reason, in order to begin to understand the transport and metabolic dynamics of oxygen in extravascular tissue, an initially avascular and ideally *in vitro* experimental model is desirable. For this work, we have studied oxygen diffusion and consumption using multicellular tumour spheroids (MTS), as these fit both criteria.

### 1.1. Multicellular tumour spheroids

Tumour spheroids are a bridge between standard two-dimensional monolayer cultures and *in vivo* models. As tumours, spheroids are three-dimensional aggregates of cancer cells which, beyond the diffusion distance of oxygen, naturally form regions of hypoxia. Additionally, their signalling and metabolic profiles are more similar to *in vivo* cells than monolayers [14]. However, similar to monolayers, spheroids are relatively straightforward to culture and easier to manipulate than tumours. For these reasons, spheroids have been widely used to investigate the development and consequences of hypoxia [14]. Measuring spheroid oxygen gradients *in situ* is possible using *pO*_2_-sensitive electrodes [15]. However, the physical disruption of the three-dimensional environment by the electrode itself can perturb the internal oxygen gradient. Oxygen-sensitive markers, such as EF5 and pimonidazole, are widely employed to detect hypoxia both clinically and in preclinical models, for example spheroids [16,17], without perturbing the cells. In this work, we will use EF5 to determine the extent of hypoxia in MTS owing to its well-defined oxygen-dependent kinetics [18].

Early interest in MTS began in the late 1970s. Spheroid growth has been examined by several authors [19–24]; in the formulation by Conger & Ziskin [25], spheroids are modelled as growing exponentially and then approximately linearly, achieving an approximately constant viable rim thickness. Mueller-Klieser [26] derived a semi-analytical piecewise model of oxygen distribution that considers the oxygen gradient subject to various boundary conditions for EMT6/Ro cells. This and its variations [27] provided an effective way to estimate the O_2_ distributions using oxygen probes and a series of equations for the different spheroid regions. The model is useful where the boundaries are clearly known but is not in itself predictive of how and where these boundaries occur, nor of the limits of these regions. Extensions of this model have been used in photodynamic formulations [28] to estimate the threshold dose for the production of reactive oxygen singlets.

Of late, there has been renewed interest in tumour spheroids and the scope for their application has increased dramatically—spheroids have been used in radiation biology [29–31] as a means to test fractionation and other parameters in a controllable environment, in chemotherapy to act as a model for drug delivery [32–35] and even to investigate cancer stem cells [36]. Cancer spheroids have also shown potential as a model for exploring FDG-PET dynamics [37] in solid tumours. In all these applications, the diffusion of oxygen and the rate at which it is consumed plays a large role and better understanding of these dynamics would be of considerable benefit. To robustly investigate oxygen consumption in MTS, we derive an analytical model which describes oxygenation through a spheroid and explicitly predicts the consumption rate for a given spheroid, as well as a prediction of the extent of the hypoxic, necrotic and proliferating regions. This model is validated here using stained cross sections of DLD1 tumour (human colorectal cancer) spheroids.

## 2. Model derivation

**Figure 1.**
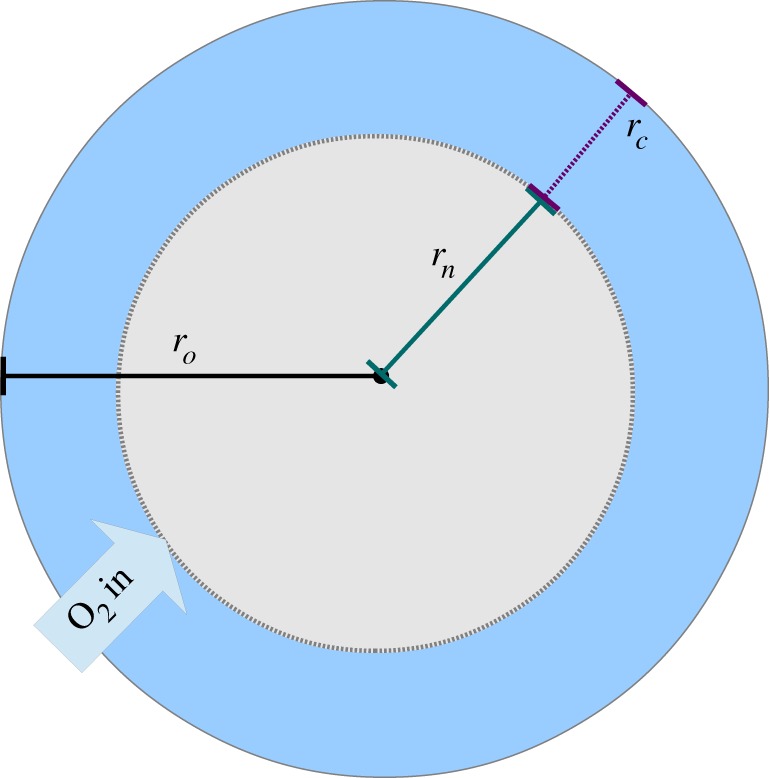
Cross section of a tumour spheroid of radius *r_o_*. Oxygen partial pressure is non-zero in the region *r_c_*. This region comprises all viable cells both hypoxic and oxic. Oxygen cannot penetrate into region *r_n_*, which is anoxic. (Online version in colour.)

Consider the diffusion equation, which relates the change in oxygenation with time to the spatial distribution. This is written as2.1
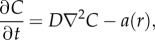
where *D* is the diffusion constant of the tissue, *C* the volume of oxygen per unit mass and *a*(*r*) the oxygen consumption rate at a point *r*. In previous literature on this subject, the quantity *C* has been defined as a concentration but this can lead to dimensional inconsistencies. In this work, we derive a robust relationship between the partial pressure and *C*, by the manipulation of Henry's law. The oxygen supply is assumed to be in steady state, and the tissue consumes oxygen at a rate *a*(*r*). Thus, the equation can be re-written in terms of the spherical Laplacian as2.2
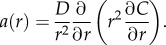
The form of *a*(*r*) is of importance in solving this equation. Initially, it is assumed that *a* is approximately constant. At the edge of the spheroid, a radius of *r_o_*, the volume of oxygen per unit mass is *C_o_*. At a distance of *r_n_* from the centre, *C* = 0 and d*C*/d*r* = 0, as illustrated in figure 1. Necrosis may set in at partial pressures slightly above zero, as cells are severely hypoxic at partial pressure of 0.8 mmHg or below [18], but as this is close to zero it does not affect the generality of the model. With these boundary conditions, we can solve equation (2.2) and write2.3
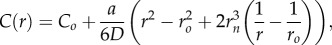
where *C* expresses the volume of oxygen gas per unit tumour mass. This is essentially the semi-analytical solution presented by Mueller-Klieser [26]. In order to manipulate this equation via Henry's law, we re-express this in terms of the mass of oxygen per unit volume. Assuming that the density of the tumour, *ρ_T_* is similar to that of water and the density of oxygen gas 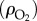 is 1.331 kg m^−3^, then we can convert this by simply multiplying *C* by 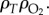 Henry's law constant *K* varies with temperature [38], and for oxygen gas at human body temperature, it may be expressed as 2.2779 × 10^−4^ m^3^ mmHg kg^−1^. Setting *Ω* = 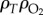
*K* = 3.0318 × 10^7^ mmHg kg m^−3^, then equation (2.3) can be expressed in terms of partial pressure as2.4
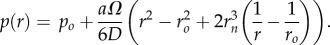
We can further extend the model to a fully analytical form by deriving an expression for *r_n_*, the anoxic limit. By definition, *C*(*r_n_*) = *p*(*r_n_*) = 0. As *r_o_* = *r_n_* + *r_c_*, equation (2.3) may be rearranged to yield a third-order polynomial equation that characterizes the spheroid.2.5
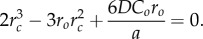

There must also be a certain spheroid size where the partial pressure of oxygen just reaches zero at the centre, which is the greatest radius the spheroid can obtain and still be entirely viable. If we define this radius as the ‘diffusion limit’ *r_l_*, then solving equation (2.6) for *r_c_* = *r_o_* = *r_l_*, this is simply2.6
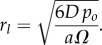


The general solution to the identity in equation (2.5) can be solved analytically with trigonometric identities. If we define *χ* as a function of *r_o_* so that2.7
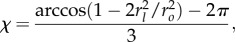
then the thickness of the viable region, *r_c_* and the anoxic region *r_n_* are given simply by2.8
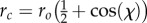
and 2.9
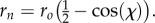
The novel result of this analysis is an expression that explicitly enables us to find the viable rim and anoxic regions in a spheroid at any stage of growth from first principles. This allows some interesting insights into spheroid growth. Consider *r_l_* as defined in equation (2.6); this can be thought of as the radius for a spheroid where the oxygen partial pressure at the centre is exactly zero. It is important to note that *r_l_* does not depend on the size of the spheroid. The limit of the viable rim, *r_c_*, can be found by taking the derivative of equation (2.5) with respect to the spheroid radius *r_o_*. This is simply2.10
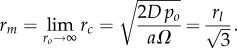


The implication of this is that the viable rim thickness decreases as the spheroid grows in size, tending towards this limit if the consumption stays approximately constant. The maximum thickness of the viable region is then *r_c_* = *r_l_* and this decreases asymptotically towards the limit *r_c_* = *r_m_* as the spheroid grows. Some previous models for MTS have assumed a constant thickness for the viable rim [25]. A growth analysis by Burton [19] suggested that for a spherical tumour with central necrosis, the rim thickness should tend asymptotically towards 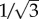 of the diffusion limit that can be reached without necrosis, which is in agreement with the form we have derived. The quantity *r_m_* is similar to the one-dimensional diffusion limit derived for a cylindrical geometry by Secomb *et al.* [39]. This new formulation makes three distinct and testable predictions, which are

(1) the diffusion limit *r_l_* is inversely related to the consumption rate and is independent of spheroid radius;
(2) the oxygen consumption rate and oxygen partial pressure at any point in the spheroid can be estimated once *r_l_* or spheroid boundaries are experimentally derived; and
(3) the viable rim thickness, *r_c_*, decreases towards a theoretical minimum of *r_m_* with increasing spheroid size.

There is some evidence that the consumption rate may vary with spheroid size, although the exact relationship is not entirely clear [26]. In such a case, a constant *r_l_* would hold only if the consumption rate for a spheroid of a given size can be treated as approximately constant; the current analysis can be used to investigate this, and the findings and implications of such are examined in the Results and Discussion sections of this study. A model considering Michaelis–Menten-type form for *a*(*r*) was also developed, but as the constant *k_m_* (the oxygen concentration at which the consumption rate is half its maximal value) is typically below 1 mmHg [40], this model form was found to have only negligible differences from the constant consumption form shown here, and has been omitted from this work.

## 3. Experimental method

### 3.1. Tumour spheroid growth and imaging

DLD1 (human colorectal carcinoma) cells were used to produce multicellular spheroids using the liquid overlay technique as described previously [41]. Briefly, tumour cells were seeded in 24-well plates coated with 1% (w/v) agarose, at a final concentration of 10 000 cells per well in 200 μl growth medium (10 mM glucose). After the initial 4-day coalescent period, the growth medium was changed every 2 days. At set timepoints between 4 and 17 days after initiation, spheroids were incubated with 300 μM EF5 at 37°C for 4 h. Spheroids were then fixed in 4% paraformaldehyde prior to cryopreservation. Frozen spheroids were cut into 10 μm sections via microtome, and the section corresponding to the central cut through the diameter was selected. Sections were then dual-stained for EF5, using a Cy3-conjugated antibody, Ki-67, a marker of proliferation, using an AlexaFluor 488-conjugated antibody. Slides were counterstained with the nuclear dye Hoechst 33342. Sections were imaged at 10× magnification using a Leica epifluorescent microscope. The oxygen partial pressure outside the spheroids in solution during growth was *p_o_* = 100 mmHg. The *pO*_2_ was monitored adjacent to the spheroid in 10 wells using the Oxylite oxygen sensor probe (Oxford Optronix). Although spheroids were maintained in incubators at a controlled temperature of 37.2°C, the temperature in each well was also measured using a digital thermometer (RS electronics) which indicated 36.9°C.

### 3.2. Determination of *r*_*o*_, *r*_*c*_ and *r*_*n*_

Spheroids were grown as outlined, and nine dual-stained DLD1 spheroids up to 17 days of growth inclusive were obtained for analysis. Artefacts irrelevant to the image were removed manually before automated analysis; these artefacts included parts of other spheroids in the image plane and orphaned staining molecules that had become dislodged during the sectioning process. A MATLAB script then performed a reproducible analysis on each spheroid. This consisted of the following steps:

— the image was segmented and the centroid was found;
— the radial distance from the centroid to the closest region above a certain threshold was determined for every 1° rotation. This gave the values for *r_n_*; and
— by stepping back from the image edge towards the centroid, the radial distance from the outer edge of the spheroid to the centroid was determined at 1° intervals, allowing determination of *r_o_.*

**Figure 2.**
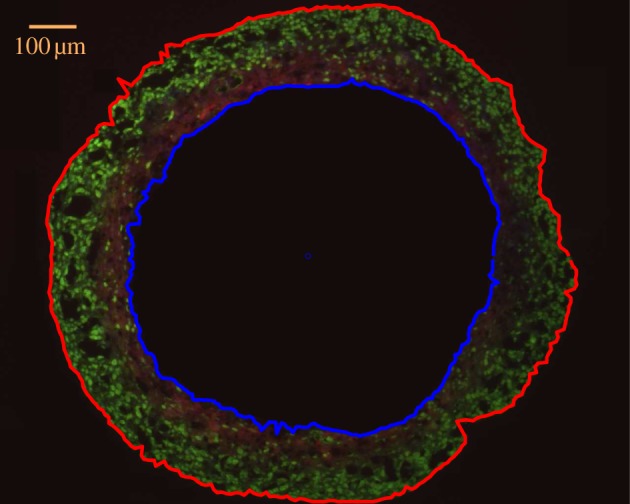
Result of applying the image analysis algorithm to an image of a day 6 spheroid. The blue boundary shows the anoxic boundary *r_n_* and the red boundary yields the spheroid radius *r_o_*, each calculated relative to the centroid in a full rotation in 1° steps around the spheroid.

The same algorithm and process was performed on each image, and the results plotted over the image to ensure accuracy. An example is shown in figure 2. This analysis ensured consistency between spheroids and reproducibility of results. Three hundred and sixty values for *r_o_*, *r_c_* and *r_n_* were averaged and the standard deviation was calculated for each spheroid image. This is important as during sectioning the spheroids can become slightly deformed and many measurements ensured the reproducibility of results as well as enabling the estimation of uncertainty. Results of segmentation were not critically dependent on choice of threshold, owing to the steep gradient in the images.

### 3.3. Determination of oxic and hypoxic interface in viable rim

**Figure 3.**
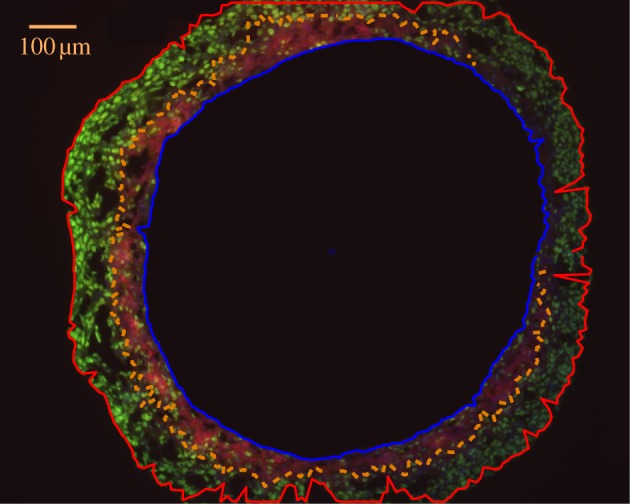
Result of semi-automatic detection on the boundary between proliferating and hypoxic regions on a day 17 spheroid. The dashed orange line indicates the best estimate of the boundary *r*_10_, relative to the spheroid centroid. When no line is present, the algorithm was unable to reliably detect the boundary.

Images were separated into their green and red colour channels to find the boundary between Ki-67 and EF5 staining. The two colour channels were both examined to find the average point of transition. The distance from the centroid to the edge of this image was calculated in 1° steps around the centroid. If the detected pixel lay beyond the spheroid boundary *r_o_* or inside the anoxic region *r_n_*, it was counted as an artefact and excluded. An example of this is illustrated in figure 3.

## 4. Model validation and testing

### 4.1. Determining *r_l_*

The diffusion distance *r_l_* can be estimated experimentally by combining and re-arranging equations (2.5) and (2.6), yielding an equivalent expression for this value of4.1
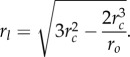
Using the image analysis technique previously outlined, it is possible to measure the spheroid radius *r_o_* and the thickness of the viable region *r_c_* for any spheroid and *r_l_* can be readily computed through equation (4.1). If the oxygen consumption rate and diffusion constant stay approximately constant at all stages of growth, then values for *r_l_* should also remain approximately constant regardless of spheroid dimensions. Thus, a value for *r_l_* can be determined with the data from each image to determine whether this relationship holds.

### 4.2. Hypoxic and oxic interface

The approximate consumption constant *a* can be estimated by manipulating equation (2.6) which would yield4.2
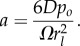
For this work, we have assumed the oxygen diffusion constant *D* is close to that of water (*D* = 2 × 10^−9^ m^2^ s^−1^) [13]. The viable regions, *r_c_*, comprise both proliferating and hypoxic cells stained, respectively, as Ki-67 staining (green) and EF5 staining (red). The point at which these boundaries meet is the threshold level for hypoxia binding. By manipulating equation (2.4) and finding the solution to the resulting equation, it is possible to find the distance *r_p_* that corresponds to any given oxygen partial pressure *p*. This is4.3
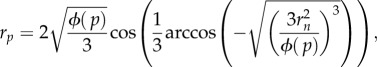
where *ϕ*(*p*) is given by4.4
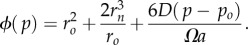
EF5 binding occurs maximally in the presence of hypoxia at partial pressures below 10 mmHg [18]. Thus, we may assume that the partial pressure at the interface between the proliferating and hypoxic regions is approximately 10 mmHg. The distance from the spheroid centre at which this transition occurs can be estimated from the images and compared to the model prediction from equations (4.3) and (4.4) for *p* = 10 mmHg, using the experimentally obtained value for consumption rate *a*. These can be compared in order to investigate and validate the model and estimate its predictive power.

### 4.3. Variation of *r_c_* with *r_o_*

The model predicts that the viable rim thickness of the spheroid will tend to decrease with the growth of the spheroid towards the limit *r_m_*.

## 5. Results

### 5.1. Model validation and determination of *r*_*l*_

**Figure 4.**
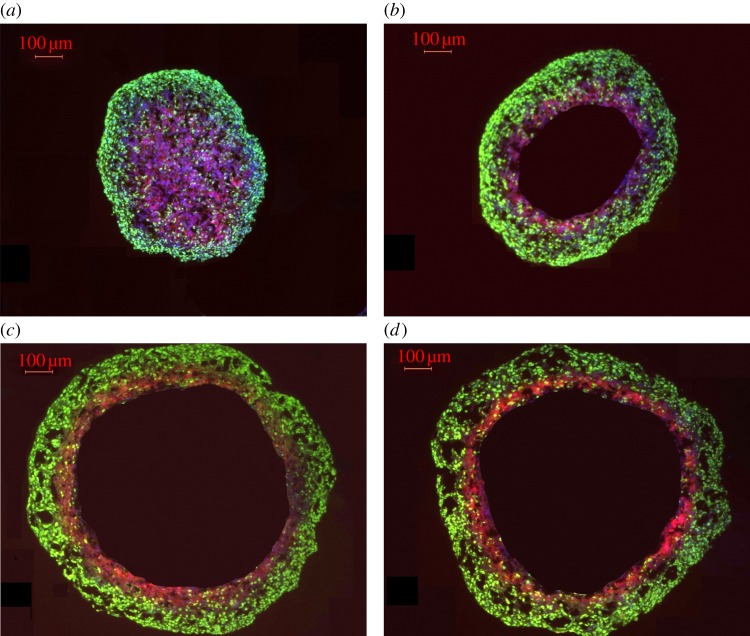
Development of hypoxia and anoxia in tumour spheroids. Tissue sections taken from tumour spheroids grown over 17 days were stained for the proliferation marker Ki-67 (green) and hypoxia (red). A distinct progression was observed: (*a*) day 4 of growth, with central hypoxia; (*b*) day 6 with beginnings of an anoxic core; (*c*) day 15 of growth, with distinct core; (*d*) day 17 of growth, distinct core and the degradation of spheroid integrity apparent.

**Figure 5.**
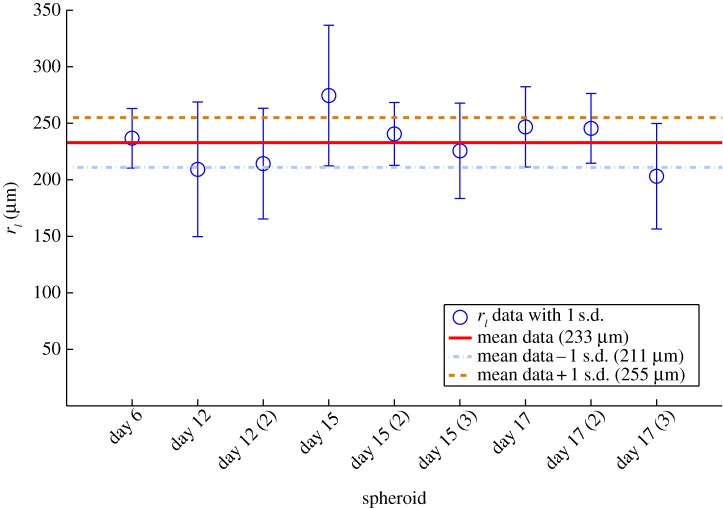
Experimentally derived value for *r_l_*, the spherical oxygen diffusion limit, for a range of different spheroids, obtained by the manipulation of equation (4.1) and applying experimentally measured values of *r_o_* and *r_n_*. The solid line shows mean value and dotted lines show 1 s.d. above and below this. (Online version in colour.)

Examples of stained spheroid sections are shown in figure 4 and in the electronic supplementary material. Three hundred and sixty measurements per spheroid over nine different spheroids were obtained, yielding values for *r_o_*, *r_c_* and *r_n_*. Using equation (4.2), a single value of *r_l_* was obtained for each spheroid. These values are shown in figure 5, with bars of ±1 s.d. shown for all spheroids. Despite the significant difference in spheroid size, quality and age, the derived value was reasonably constant at 233 ± 22 μm. The approximately constant *r_l_* gives rise to an approximately constant estimate for *a* for all spheroids studied through the use of equation (4.2), as given in table 1, with standard deviation of ±1.4 × 10^−7^ m^3^ kg^−1^ s^−1^, or approximately 1.82 ± 0.35 × 10^−3^ mol kg^−1^ min^−1^.

**Table 1.** *r_l_* and consumption rate.

value	derived value for *a*
233 μm (mean value)	7.29 × 10^−7^ m^3^ kg^−1^ s^−1^
211 μm (mean − 1 s.d.)	8.89 × 10^−7^ m^3^ kg^−1^ s^−1^
255 μm (mean + 1 s.d.)	6.09 × 10^−7^ m^3^ kg^−1^ s^−1^

### 5.2. Hypoxic and oxic region

**Figure 6.**
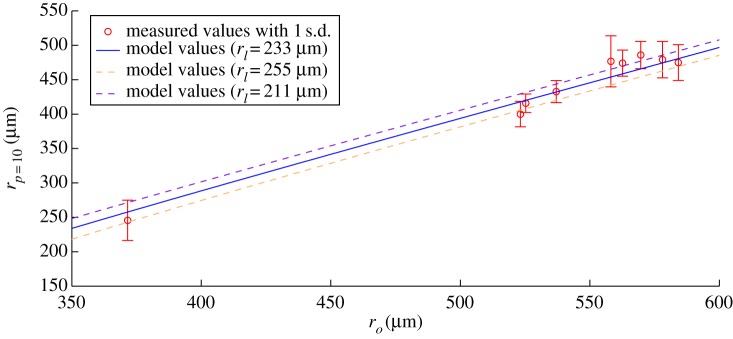
Model simulation versus measured values for *r_p_*_=__10_, the radius at which partial pressure of oxygen *p* = 10 mmHg, obtained by manipulation of equations (4.3) and (4.4). The solid line indicates the value of *r_p_*_=__10_ predicted by the model using an experimentally derived value for the spherical oxygen diffusion distance *r_l_*, as a function of spheroid radius *r_o_*. The dotted lines indicate the predicted value of *r_p_*_=__10_ allowing all for 1 s.d. on the assumed spherical oxygen diffusion distance *r_l_*, (±22 μm). (Online version in colour.)

The distance from the centre of the spheroid to the interface between the hypoxic and proliferating region was denoted *r_p_*_=__10_. Using the derived value for *a* given in table 1, solutions to the model (equation (2.4)) for an oxygen partial pressure of 10 mmHg were obtained. These are compared to the data obtained experimentally and depicted in figure 6. Error bars of ±1 s.d. are shown along the vertical (*r_p_*_=__10_) axis. For clarity, the uncertainties on the *x*-axis (*r_o_*) are not shown on this figure. These range between 16 and 30 μm for the various data points. A regression analysis on the solid line data shown and the measured points yields a co-efficient of determination of *R*^2^ = 0.9635 for *r_l_* = 233 μm, indicating a high level of agreement between the model and experimental data.

### 5.3. Variation of *r_c_* with *r_o_*

**Figure 7.**
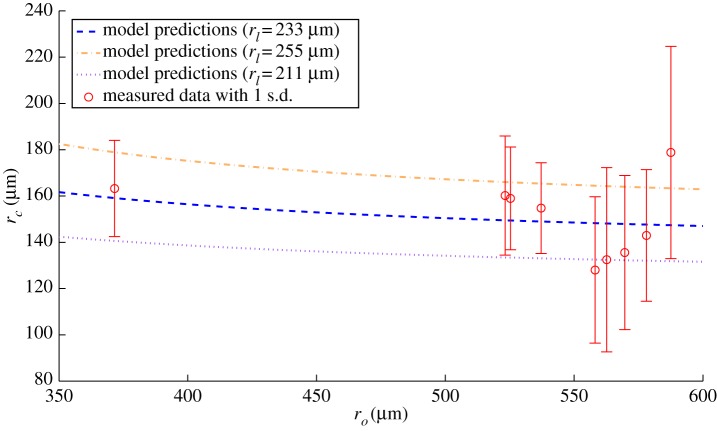
Variation of viable rim thickness *r_c_* with spheroid radius *r_o_*—model values for *r_l_* = 232 ± 22 μm is shown. All measured points are shown with error bars of 1 s.d. (Online version in colour.)

The model predicts a small variation of the viable radius with increasing spheroid radius; experimental data and model predictions are shown in figure 7 with the vertical bars representing 1 s.d. Again, the uncertainty on the *x*-axis (*r_o_*) is not shown for clarity. All the data points lie within 1 s.d. of the predicted value, and almost all lie within the envelope, indicating agreement. However, it should be noted that for the range of values of *r_o_* available, the model predicts that the absolute maximum difference in *r_c_* would be only 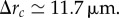 This is less than the uncertainty in the measurements for *r_o_* and *r_c_*, making conclusive validation impossible. However, all the measured data lie between the *r_l_* and *r_m_*, as predicted by the model and underlying theory, indicating that the model predictions are not inconsistent with the experimentally derived data.

## 6. Discussion

For the DLD1 spheroids studied, *r_l_* had a relatively constantly value of 233 ± 22 μm, with the low deviation indicating that the consumption rate for all spheroids studied was approximately constant at *a* = 7.29 ± 1.4 × 10^−7^ m^3^ kg^−1^ s^−1^, in good agreement with previous data from similar approaches [26]. It was important to further verify whether the model could predict the oxygen partial pressure at any point within the spheroid: as the spheroid was dual-stained, the boundary between the Ki67 and EF5 staining implied that the partial pressure at this point was approximately 10 mmHg. This could be theoretically predicted by equations (2.3) and (2.4) and compared to the value found with analysis of sectioned spheroid images. The model predictions and measured data were found to be in good agreement, with a fit of *R*^2^ > 0.96. This also provides good evidence that the constant value of *r_l_* was not merely due to chance. Finally, the prediction that *r_c_* will tend towards the limit *r_m_* as *r_o_* increases was also tested. This was done by using the experimentally derived value of *r_l_* and comparing modelled model values of *r_c_* with the measured data. All of the data points investigated lay within 1 s.d. of the model predictions. However, greater resolution would be needed to confirm this trend, as the decrease in *r_c_* is difficult to observe, being less than the uncertainty in the measured values of *r_c_*. This is a challenge to overcome, because the majority of the uncertainty in *r_c_* is due to irregularities in spheroid growth or distortion induced during sectioning. In summary, the model predictions were observed experimentally, indicating strongly that the model provides a quantitative description of the spheroid growth characteristics for this cell line and that boundaries are influenced mainly by oxygen diffusion limits.

**Figure 8.**
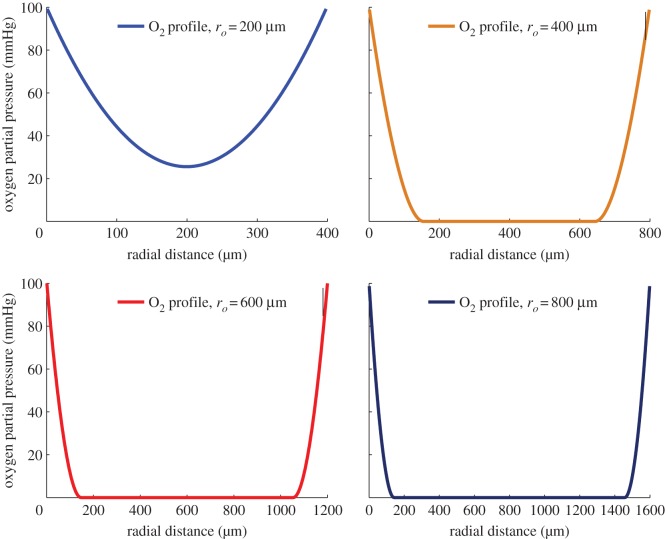
Simulated O_2_ profiles for spheroids of various sizes for *r_l_* = 233 μm. (Online version in colour.)

One advantage of the proposed model is that it allows direct prediction of the spheroid dimensions and properties from first principles, and predicts these regions well at any stage of spheroid growth. Specifically, the characteristic equation of the spheroid allows the determination of vital parameters, such as anoxic radius *r_n_* and viable rim thickness *r_c_* from the physical properties, yielding a model that is fully analytical. It also enables the prediction of oxygen profiles without invasive measurement which may perturb the oxygen gradient within a spheroid; examples of which are illustrated in figure 8. These profiles are similar in form to previously measured results [15,42] and can be matched by adjusting the parameter values.

**Figure 9.**
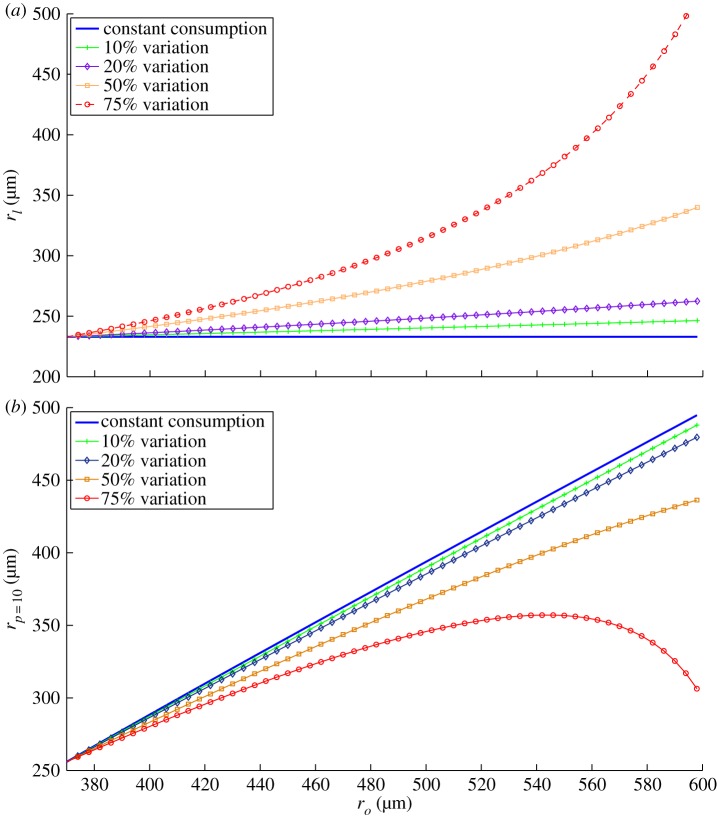
(*a*) The expected variation of *r_l_* and (*b*) expected variation of *r_p_*_=__10_ with decreasing *a*. The measured data imply heavily that the consumption was approximately constant for all spheroids in the set. (Online version in colour.)

There has been some discussion in the literature regarding whether the variation in consumption rate changes as spheroids grow. Previous work [26] used oxygen probes *in situ* to estimate the partial pressure gradient and from this estimated the value of *a* for 15 EMT6/Ro spheroids; while the standard deviation of this dataset was only 10% of the mean value, early growth spheroids appeared to have consumption rates markedly above average. Consumption has been estimated to vary up to 50% with cell size in some cell lines, while minimal variation is seen in other cell lines [43]. For this analysis, the variation in consumption rate should have quite an effect on the spheroid properties; as 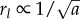, this indicates a slowly increasing *r_l_* as consumption decreases. Such a scenario is depicted in figure 9*a* with simulated drops in the consumption rate of 10, 20, 50 and 75% across the dataset contrasted with constant consumption. This trend was not observed in our data. Similarly, a large decrease in *a* would have an equally striking impact on the interface measurements. This is depicted in figure 9*b*. Simulated curves can be contrasted to the measured data to determine how good the fits are and the results of this are given in table 2. The fit to measured values become increasingly poor as the variation is increased, and markedly less linear, yielding a negative *R*^2^ at 75% variation. These fits and figures indicate strongly that appreciable variation in consumption rate was not seen for the cell line, growth conditions and size range of spheroids analysed here.

**Table 2.** *R*^2^ fits for *r_p_*_=__10_ data.

variation of *a* across dataset	goodness of fit with data
constant	0.9635
10% variation	0.9481
20% variation	0.9160
50% variation	0.5724
75% variation	−1.0898

There are several reasons that might explain this discrepancy; firstly, the EMT6/Ro and DLD1 cell lines have different growth kinetics. Owing to the rapid growth of DLD1 spheroids, all of our datasets were taken at points where *r_o_* > *r_l_* with central anoxia; by contrast, the analysis of the EMT6/Ro data suggests that many of the spheroids analysed in their work had *r_o_* < *r_l_*, without central anoxia, making direct comparison between the two approaches difficult. Early work on tumour spheroids suggested that at very early growth their consumption rate is high, rapidly dropping to a constant level as spheroids grow [44]. Other authors [37] treat the oxygen consumption as relatively constant at normal oxygen tensions, falling rapidly at extremely low partial pressures. In both of these cases, the value *a* in this work can be considered as the plateau oxygen consumption rate under normal oxygen pressures. This is an avenue worthy of further exploration, and more investigations between different cell lines would be required to answer this with certainty. Finally, the methodology of this and previous approaches is quite different, as the current method takes a first-principles approach and validates using sectioned stained images.

The oxygen consumption of a two-dimensional array of cells was measured by using a Seahorse Extracellular Flux Analyser (Seahorse Biosciences, Billerica, CA, USA). This indicated the quantity of oxygen consumed by DLD1 cells (moles of oxygen per minute per 80 000 cells) was 250 pmol min^−1^ per 80 000 cells. This implies that each cell consumes 1.25 × 10^−18^ m^3^ of oxygen every second. To see how this compares to our values of *a*, we first assume that these consumptions are equivalent and that cells have the same density as water. From this, an estimate of cell volume can be obtained. Under the assumption of these cells being approximately spherical, this yields a cellular radius of 7.42 ± 0.47 μm. To investigate how accurate this estimate was, individual DLD1 cells were suspended and their area were measured under a microscope. This yielded a mean value for suspended cell radius of 6.97 ± 0.68 μm, which is in very good agreement with estimates. The estimate of *D* is also a potential confounding factor; the estimate of *a* is dependent on *D* by equation (2.6) and this likely varies among different tumour types. However, as other authors have noted [13,37,45], it is likely to be close to water and as such the value used here is a reasonable estimate, though better determination for a given spheroid type would improve accuracy. The value of *p_o_* is another potentially confounding factor—previous investigations [26] have indicated that a diffusion depleted zone can exist up to 100 μm surrounding the spheroid where the oxygen partial pressure is below that measured in the bulk medium. To circumvent this, oxygen probe measurements were taken as close as possible to the spheroid edge and this value is taken as *p_o_*. The value for *r_p_*_=__10_ is dependent on this measurement as seen in equation (4.4), and the good agreement between measured and predicted *r_p_*_=__10_ indicates that this value was a realistic estimate. As 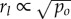, better determination of *p_o_* yields a more accurate estimation of *r_l_* and consumption rate. While the literature indicates that EF5 binds between 0.8 and 10 mmHg, a sensitivity analysis was performed to estimate the resultant errors if the upper limit for EF5 binding was significantly under- or overestimated; even at 20% error (upper limit for binding occurring at 8–12 mmHg) the projected *r_p_*_=__10_ difference was only 4.5 μm, indicating that potential errors introduced by binding sensitivity are small and effectively negligible.

The current model does not consider the glucose consumption, focusing instead on the oxygen diffusion. The evidence presented here indicates that for the case of DLD1 spheroids, oxygen diffusion is by far the dominating factor in spheroid growth. A variation of the model was derived for oxygen consumption that varied with Michaelis–Menten kinetics, but this was found not to differ markedly from the model presented here for the low values of *k_m_* [40,46] and is consequently not detailed here. The model is time independent, but in theory it could be extended to factor in growth rate. This is beyond the scope of the present work, but despite this time independence, the model does make useful predictions about growth and growth limits. Moreover, it allows the quantification of oxygen diffusion through avascular tissue, including oxygen distributions and likely patterns of hypoxia and proliferation. While the method outlined in this work allows calculation of the anoxic boundary, the relationship between hypoxia and necrosis is complicated. Literature reviews [47] suggest that there are cases when hypoxia and necrosis coincide, cases where necrosis sets in after hypoxia and even cases where necrosis can precede hypoxia; although this behaviour is not seen with the cell line and experimental conditions in this work, and the scope of our modelling is limited to the proliferation, hypoxia and necrosis patterns shown. For cell lines that do not readily aggregate in spheroids, alternative modalities such as multicellular layers (MCL) have shown promise in investigating oxygen distribution with a one-dimensional diffusion model, but it is currently difficult to determine predictive boundaries with this method [48]. The spheroid results presented serve as an important validation of the underlying theory, and future work will include applying similar models to histological measurements of oxygen in tissues with known microvessel vascular maps, and contrasting this with macroscopic imaging to better quantify likely hypoxia distribution in tumours, and therefore better inform strategies for dose painting in radiotherapy.

## 7. Conclusion

We present a method for estimating oxygen diffusion and consumption rate in MTS, using an analytical solution of the spherical reaction–diffusion equation. The advantage of this model is that it provides a prediction of the extent of viable, hypoxic and anoxic regions in a spheroid. It also predicts analytically how these regions will change with increasing spheroid size. We have shown that model predictions of the spherical oxygen diffusion limit, the hypoxic boundary and the viable rim thickness are all in agreement with experimental measurement using stained sections of DLD1 spheroids, and the estimated oxygen consumption rate was in agreement with measured values.
